# Modelling and identification of characteristic kinematic features preceding freezing of gait with convolutional neural networks and layer-wise relevance propagation

**DOI:** 10.1186/s12911-021-01699-0

**Published:** 2021-12-07

**Authors:** Benjamin Filtjens, Pieter Ginis, Alice Nieuwboer, Muhammad Raheel Afzal, Joke Spildooren, Bart Vanrumste, Peter Slaets

**Affiliations:** 1 Intelligent Mobile Platform Research Group, Department of Mechanical Engineering, KU Leuven, Andreas Vesaliusstraat 13, 3000 Leuven, Belgium; 2 eMedia Research Lab/STADIUS, Department of Electrical Engineering (ESAT), KU Leuven, Andreas Vesaliusstraat 13, 3000 Leuven, Belgium; 3 Research Group for Neurorehabilitation (eNRGy), Department of Rehabilitation Sciences, KU Leuven, Tervuursevest 101, 3001 Heverlee, Belgium; 4 Faculty of Rehabilitation Sciences, REVAL - Rehabilitation Research Center, Hasselt University, Agoralaan Building A, 3590 Diepenbeek, Belgium

**Keywords:** Explainable artificial intelligence, Convolutional neural networks, Freezing of gait, Parkinson’s disease, Gait analysis

## Abstract

**Background:**

Although deep neural networks (DNNs) are showing state of the art performance in clinical gait analysis, they are considered to be black-box algorithms. In other words, there is a lack of direct understanding of a DNN’s ability to identify relevant features, hindering clinical acceptance. Interpretability methods have been developed to ameliorate this concern by providing a way to explain DNN predictions.

**Methods:**

This paper proposes the use of an interpretability method to explain DNN decisions for classifying the movement that precedes freezing of gait (FOG), one of the most debilitating symptoms of Parkinson’s disease (PD). The proposed two-stage pipeline consists of (1) a convolutional neural network (CNN) to model the reduction of movement present before a FOG episode, and (2) layer-wise relevance propagation (LRP) to visualize the underlying features that the CNN perceives as important to model the pathology. The CNN was trained with the sagittal plane kinematics from a motion capture dataset of fourteen PD patients with FOG. The robustness of the model predictions and learned features was further assessed on fourteen PD patients without FOG and fourteen age-matched healthy controls.

**Results:**

The CNN proved highly accurate in modelling the movement that precedes FOG, with 86.8% of the strides being correctly identified. However, the CNN model was unable to model the movement for one of the seven patients that froze during the protocol. The LRP interpretability case study shows that (1) the kinematic features perceived as most relevant by the CNN are the reduced peak knee flexion and the fixed ankle dorsiflexion during the swing phase, (2) very little relevance for FOG is observed in the PD patients without FOG and the healthy control subjects, and (3) the poor predictive performance of one subject is attributed to the patient’s unique and severely flexed gait signature.

**Conclusions:**

The proposed pipeline can aid clinicians in explaining DNN decisions in clinical gait analysis and aid machine learning practitioners in assessing the generalization of their models by ensuring that the predictions are based on meaningful kinematic features.

**Supplementary Information:**

The online version contains supplementary material available at 10.1186/s12911-021-01699-0.

## Background

Parkinson’s disease (PD) is the second most common neurodegenerative disorder, impacting over 6 million people worldwide [1]. Freezing of gait (FOG) is one of the most debilitating symptoms of PD, given that an estimated 20-60% of falls and fall-related injuries for this group can be directly attributed to this paroxysmal symptom [2, 3]. Moreover, FOG is common in PD, with approximately 70% of Parkinson’s disease patients developing FOG over the duration of the disease [4, 5]. FOG is clinically defined as a “brief, episodic absence or marked reduction of forward progression of the feet despite the intention to walk” [6]. PD patients describe freezing of gait as “the feeling that their feet are glued to the ground” [7]. PD patients with FOG have more anxiety and falls [8–12], and an overall lower quality of life [13]. Freezing episodes are most frequently provoked when traversing small spaces, during turning and gait initiation, and while dual-tasking [14, 15]. However, and especially in gait laboratories, it is common that FOG does not occur, despite providing adequate FOG-provoking conditions [15].

To date, Levodopa is the gold standard intervention for the treatment of PD. Levodopa shows a positive effect on FOG [16], with 95% of PD patients showing FOG to a lesser degree after Levodopa is administered [17]. However, the relationship between FOG and Levodopa remains complex, as Levodopa often only elicits a partial response in the more advanced stages and may even exacerbate FOG [18, 19]. Non-pharmaceutical interventions, such as sensory cueing, have shown to improve gait and reduce the severity of FOG [20–24]. The notion of sensory cueing relates to the provision of spatial (visual) stimuli to regulate stride placement and amplitude, or temporal (auditory or somatosensory) stimuli to regulate stride timing and regenerate gait. PD patients have shown to adapt to cueing, reducing the effectiveness of the intervention over time [25]. Hence, the provision of continuous stimuli carries the risk of habituation, which may also negatively impact patient compliance [26]. Furthermore, it has been suggested that the optimal cue timing is before the onset of a FOG episode, as providing cues during a FOG episode may result in cognitive overload [26, 27].

To facilitate research in on-demand preventive cueing, there is a clear need for an automated approach to objectively predict the onset of FOG [27]. Several studies have attempted to characterize and predict FOG [28–31], typically by relying on manually extracted features and traditional machine learning techniques. However, the pathophysiology of FOG is complex and characterized by highly variable gait patterns between subjects [32–34]. Moreover, FOG is characterized by several apparent gait sub-types: (1) Akinetic FOG, characterized by a complete absence of movement in the lower and upper limbs. (2) Trembling FOG, characterized by an alternating tremble of the legs at a frequency of 3 to 8hz. (3) Shuffling FOG, characterized by small shuffling steps with minimal forward displacement [16]. These characteristics make it challenging to hand-engineer features that generalize across subjects and sub-types. Therefore, there is increasing interest in Deep learning (DL) techniques to model FOG [35–40].

Owing to their large parametric space, deep learning techniques can infer relevant features directly from the raw input data, a technique called end-to-end learning [41]. However, the large parametric space has as a downside that deep learning models are considered to be black-box algorithms, i.e. there is a lack of direct understanding of the models’ ability to identify relevant features [42]. For FOG prediction, where an intervention to alleviate FOG may be triggered before an episode has visually occurred, it will be especially challenging to motivate the provision of the stimuli. This phenomenon hampers further insight into the complex characteristics that define FOG. Therefore, clinical applications tend to avoid deep learning techniques and use simpler and more interpretable techniques [43].

Interpretability methods have been developed to ameliorate this concern by providing a way to explain the predictions of black-box deep neural networks (DNN). The essential idea behind these methods is to decompose the predicted probability of a specific target into a set of attribution values, sometimes also termed “relevance scores”, to each input sample of the network [44]. The present study goes further than deep learning-based FOG prediction by presenting a two-stage pipeline consisting of: (1) a convolutional neural network (CNN) to model the characteristic kinematic features that differentiate gait cycles that directly precede FOG from their functional counterparts, and (2) layer-wise relevance propagation (LRP) [45] to interpret the trained model and visualize the features that the model perceives as important to the classification problem. LRP is a recently developed gradient-based attribution technique, that has been previously employed to explain DNN predictions in MRI-based Alzheimer’s disease classification [46], EEG classification [47], and to explain the unique characteristics of individual gait patterns [48]. To the best of our knowledge, this is the first study that applies an interpretability method in clinical gait analysis in general and FOG prediction in particular. The proposed pipeline aims to aid clinicians in explaining DNN decisions, and aid machine learning practitioners in assessing the generalization of their DNN models.

## Methods

### Subject characteristics

An existing dataset [49] of twenty-eight patients diagnosed with PD and in Hoehn & Yahr stage II or III while on medication, and fourteen healthy age-matched controls was used. The PD diagnosis was established by a movement disorders neurologist. Patients were further classified as PD with FOG, from now on called “freezers”, by the New Freezing of Gait Questionnaire [50], when they reported that they had experienced FOG in the past month after showing them a video of different types of freezing episodes, including very mild ones (NFOGQ $\ge$ 1). Patients without FOG, called “non-freezers”, reported not to have had such episodes over this period (NFOGQ = 0). Freezers who did not freeze during the actual experiments are indicated as “NoLab-freezers”. The study was approved by the local ethics committee of the University Hospital Leuven and all subjects gave written informed consent. The clinical characteristics of the three subject groups are presented in Table 1.

**Table 1:** Subject characteristics of the fourteen healthy controls (controls), fourteen PD patients without FOG (non-freezers), and fourteen PD patients with FOG (freezers) in terms of mean ± SD as measured during the ON-phase of the medication cycle

	Controls	Non-freezers	Freezers
Age (years)	65.2 ± 6.8	66.7 ± 7.4	68.6 ± 7.4
Disease duration (years)		7.8 ± 4.8	9.0 ± 4.8
UPDRS III [81]		34.4 ± 9.9	37.9 ± 14.0
H&Y [82]		2.4 ± 0.3	2.5 ± 0.5

**Table 2:**
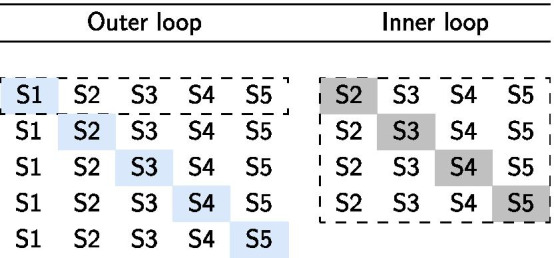
Visual overview of the nested leave one subject out cross validation

For simplicity, the visualization is given for five subjects (S1–S5). The dashed lines are added to denote that the visualization is limited to a single iteration of the outer loop, visualizing the tuning procedure for left-out test subject S1. For this single iteration of the outer loop, subject 1 (S1) is left-out as a true holdout set. The remaining subjects (S2–S5) are utilized to optimize the network parameters in the inner loop. For each hyperparameter set, the inner loop computes the prediction accuracy by iteratively using each inner loop subject as a holdout validation set. The hyperparameter set that results in the highest average accuracy on the inner loop subjects is utilized to train a model on all subjects of the inner loop (S2–25). This trained model is utilized to compute the metrics and explanations of the left-out test subject (S1). This process is repeated for all subjects

### Procedure

**Fig. 1:**
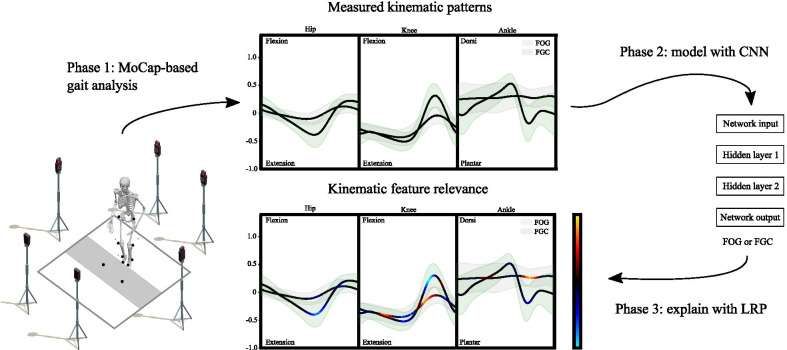
Visualization of the proposed methodology. The proposed methodology consists of two-stages (1) a convolutional neural network (CNN) to model the dramatic reduction of movement present before a freezing of gait (FOG) episode (Phase 2), and (2) layer-wise relevance propagation (LRP) to interpret the underlying features that the CNN perceives as important to model the pathology (Phase 3). The CNN was trained with the sagittal plane kinematics as recorded by a motion capture system (Phase 1). The figure illustrates the benefit of interpretation in a deep learning framework

Gait analysis was performed using an eight-camera Vicon 3D motion analysis system recording at a sampling frequency of 100Hz (Fig. 1: Phase 1). Thirty-four retro-reflective markers were placed on anatomical landmarks according to the full-body plug-in-gait model [51, 52]. Two retro-reflective markers placed .5 m from each other indicated where subjects either had to (1) walk straight ahead, (2) turn 180$^{\circ }$left, (3) turn 180$^{\circ }$right, (4) turn 360$^{\circ }$left, or (5) turn 360$^{\circ }$right. The five experimental conditions were offered randomly and performed with or without a verbal cognitive dual-task, namely the color classification task [53, 54]. All experiments were done during the off-state of the subjects’ medication cycle (after an overnight withdrawal of their normal medication intake), except for clinical testing which was conducted ON-medication [49].

**Fig. 2:**
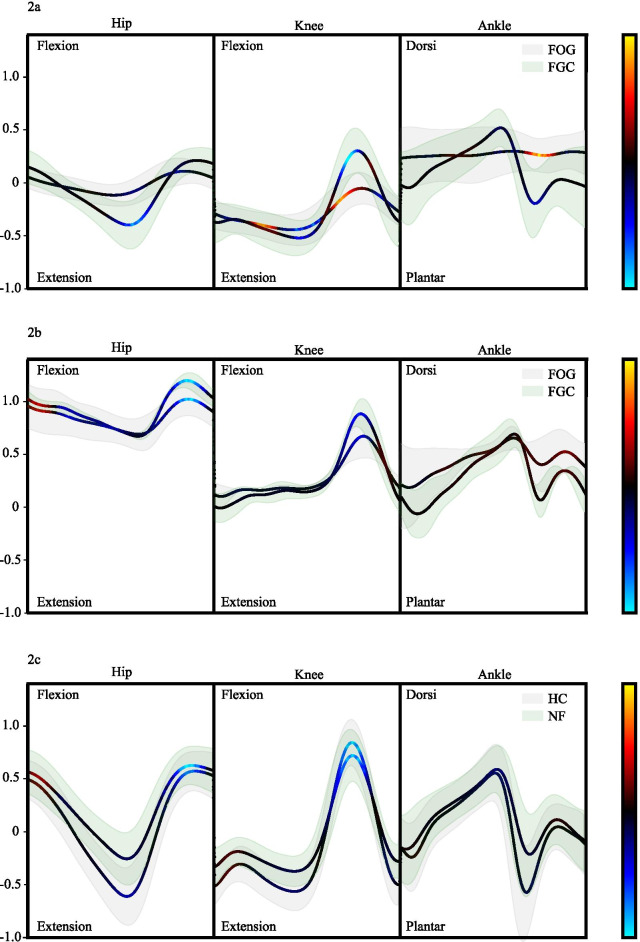
Mean and standard deviation of the hip, knee, and ankle joint trajectories in the sagittal plane for six of the seven freezers who experienced FOG during the protocol (**a**), with the excluded subject discussed separately (**b**), and the fourteen non-freezers and fourteen healthy control subjects (**c**). The joint trajectories are colorized with the relevance map (heatmap) $\sum _{x} R_{x}^{(1)}$ using $\epsilon$-LRP. To ensure an equal contribution, six strides (three pre-FOG and three FGC) are used of each freezer, with exception of subject seven who only froze once. For the non-freezers (NF) and healthy control (HC) subjects, all 2421 and 2258 strides were used. For the attribution plots of the freezers (**a** and **b**), the error clouds depict the standard deviations of the pre-FOG trajectories (gray) and FGC trajectories (green). For the attribution plots of the NF and HC (**c**), the error clouds depict the standard deviations of NF trajectories (green) and HC trajectories (gray). Positive relevance (red) indicates contribution to FOG, while negative relevance (blue) indicates contribution to FGC

Two researchers, blinded for NFOG-Q score, visually detected all FOG episodes. The onset of FOG, defined as the start of delayed knee flexion, was detected by visual inspection of the knee-angle data (flexion-extension) in combination with the Vicon 3D images. Termination of FOG was determined at the time point when at least two consecutive movement cycles were regained [55].

The last complete gait cycle before the onset of the freezing episode was chosen as the positive class. To obtain representative data for functional gait, each pre-FOG cycle was matched with one functional gait cycle (FGC) of the same subject (if possible) and one FGC of one of the seven “NoLab-freezers” that did not freeze during the experiments. For the pre-FOG and FGC matching, preference was given to functional strides that occurred during the same experimental protocol and within the same section of the turning radius that was utilized to elicit the FOG episode. This matching protocol was not always possible if, for example, a patient was unable to complete a certain experiment without freezing. To preserve class imbalance if no identical matching FGC could be found, the mismatched FGCs were left included in the training dataset, however, the mismatched pairs were excluded during the attribution analysis. This protocol allowed us to control for class imbalance while ensuring that the variability of all fourteen freezers remained present in the dataset. To prevent human bias and error, our data-driven model [35] was used to automatically extract the gait cycles. It should be noted that the gait cycles termed as “functional gait” were extracted from all fourteen freezers. These functional gait cycles thus included highly impaired movement and it cannot be ensured that had the experiment continued would not have amounted to a freezing episode. However, this more conservative protocol allows the network to model the characteristic movement that precedes FOG, rather than general movement that differentiates freezers from non-freezers.

### Data preprocessing and problem formulation

The balanced dataset of pre-FOG and functional gait cycles $[\{X_1, Y_1\}, \{X_2, Y_2\}, \dots , \{X_M, Y_M\}]$ is a collection of M pairs $\{X_i, Y_i\}$, where each gait cycle $X_i$ is a collection of joint trajectories and $Y_i$ its respective label. Each gait cycle was low-pass filtered with a cut-off frequency of 7 Hz [56] using a forward-backward fourth-order butter-worth filter and was resampled to 101 samples such that each sample corresponds to one percent of the gait cycle. Each input signal $X_i = x_{1}, x_{2}, \dots , x_{101}$ thus consists of 101 real-valued joint trajectories, where the joint trajectories $x \in \mathbb {R}^{1\times 3}$ are composed of the sagittal plane kinematics $x_h, x_k, x_a$, respectively the hip, knee, and ankle components. To ensure an equal contribution of all joint trajectories [57], each joint trajectory $x_i$ was individually re-scaled to a range of $[-1,1]$. $Y \in \mathbb {R}^{M\times 2}$ is the one-hot encoded label vector, where each element $Y_i \in \{0,1\}$, is equal to 1 if the gait cycle $X_i$ is preceding a FOG episode and 0 if it is a functional gait cycle. The goal of a deep learning model is to classify the multivariate input signal $X_i \in \mathbb {R}^{101\times 3}$ into its corresponding label $Y_i$ (Fig. 1: Phase 2).

### Model definition

Deep Neural Networks (DNNs), such as Convolutional Neural Networks (CNNs), have shown state of the art results in time series classification [58]. A CNN [59] consists of altering convolutional and pooling layers and comprises three phases. In the first phase, the input signal is convolved in a convolutional layer with a set of filters, where each filter is defined by a weight matrix *W* and bias *b*. These convolutions consist of element-wise multiplications and summations of the input signal and have an interesting property called parameter sharing, i.e. the same convolution (filter values *W* and *b*) is used for all time samples of the input signal [60]. This property enables a CNN to learn features that are invariant across the time dimension [58]. In the second phase, the output of the convolution is passed through a non-linear activation function. In the third phase, the non-linearity is followed by a local pooling layer to reduce the dimensionality of the convolutional layer output.

The result is a *p*-dimensional feature vector, where *p* is equal to the number of filters. The feature vector is fed into a global average pooling layer [61], which drastically reduces the number of parameters compared to a traditional summation. The pooled features are then transformed to predictions over the output label through a softmax activation function. To improve regularization, dropout [62] along with max-norm regularization, and a sigmoidal decaying learning rate was used.

During training, the weights are optimized to minimize the error between the model prediction $\hat{Y}_{i}$ and the observed data $Y_{i}$, defined as the loss function. To account for class imbalance, a weighted categorical cross-entropy loss was used [60]:

1 $$\begin{aligned} L(Y_{i}, \hat{Y}_{i}) = -\sum _{i} \alpha _{i} Y_{i}log\hat{Y}_{i}, \end{aligned}$$

where *L* is the loss and $\alpha _{i}$ the weighing factor of class *i*.

As a simple baseline, a support vector machine (SVM) [63, 64] with a linear kernel was implemented. For the simple baseline, the Linear Support Vector Classifier (LinearSVC) of the scikit-learn toolbox [65] was used with a regularization parameter C of 0.01.

### Model selection

To find a good set of hyperparameters, a recently proposed Bayesian optimization algorithm was used [66]. For a complete overview of the optimized hyperparameter space, the reader is referred to Table 1 in Additional file 1: Table S1. Model selection and training was done by following a nested cross validation approach, with training and validation folds split by subject, as formalized in Table 2. To assess generalization of the model to a different cohort of subjects, a pre-trained model on the fourteen freezers was used to predict the gait cycles of the fourteen non-freezers and fourteen healthy control subjects. Since the dataset consists out of balanced pre-FOG and functional pairs for the PD patients with FOG that froze during the experiments and solely functional cycles for the NoLab-freezers that did not freeze during the experiments, the results were summarized in terms of accuracy:

2 $$\begin{aligned} Accuracy = \frac{Number \, of \, correct \, predictions}{Number \, of \, all \, predictions} \, \% \end{aligned}$$

For the fourteen freezers, the models’ predictions were additionally summarized with the positive and negative predictive values (PPV and NPV), the sensitivity, and the specificity, defined as:

3 $$\begin{aligned} PPV= & {} \frac{Number \, of \, true \, positives}{Number \, of \, true \, positives + \, false \, positives} \, \% \end{aligned}$$

4 $$\begin{aligned} NPV= & {} \frac{Number \, of \, true \, negatives}{Number \, of \, true \, negatives + \, false \, negatives} \, \% \end{aligned}$$

5 $$\begin{aligned} Sensitivity= & {} \frac{Number \, of \, true \, positives}{Number \, of \, true \, positives + \, false \, negatives} \, \% \end{aligned}$$

6 $$\begin{aligned} Specificity= & {} \frac{Number \, of \, true \, negatives}{Number \, of \, true \, negatives + \, false \, positives} \, \% \end{aligned}$$

To determine if the differences in predictive performance between the two evaluated methods are statistically significant, a McNemar’s test was performed [67]. The McNemar’s test, sometimes also called a “within-subjects chi-squared test”, is a non-parametric statistical test for paired nominal data that can be used to compare the performance of two classifiers [68]. McNemar’s test evaluates the null hypothesis that there is no difference in the classification performance of the two methods. For the statistical evaluations, the significance level was set to 95%, which means that the differences are considered statistically significant if the calculated p-values are lower than 0.05.

### Model interpretation

Layer-wise Relevance Propagation (LRP) [45] was used to improve transparency and provide insight into the predictions of the DL model (Fig. 1: Phase 3). LRP is a commonly used attribution technique that decomposes the prediction of a particular output $Y_i$, computed over a gait cycle $X_i$, down to relevance scores of each input sample. Formally, LRP computes the relevance by back-propagating over the following equation:

7 $$\begin{aligned} R_{i}^{(l)} = \sum _{j} \frac{z_{ij}}{\sum _{i'} z_{i'j}} R_{j}^{(l+1)} \quad \text {with}\quad z_{ij} = x_{i}^{(l)} w_{ij}^{(l,l+1)}, \end{aligned}$$

where $R_{i}^{(l)}$ is the relevance of unit *i* of layer *l*. This decomposition results in a relevance map (heatmap) $\sum _{x} R_{x}^{(1)}$, which demonstrates the importance of each input sample $x_i$ to the prediction of the output. This study uses the epsilon variant of LRP ($\epsilon$-LRP), as implemented in [44]:

8 $$\begin{aligned} R_{i}^{(l)} = \sum _{j} \frac{z_{ij}}{\sum _{i'} z_{i'j} + \epsilon \, sign(\sum _{i' z_{i'j}})} R_{j}^{(l+1)}, \end{aligned}$$

where the term $\epsilon$ is added to the denominator of Equation 10 to avoid numerical instabilities. For a theoretical deduction of LRP the reader is referred to [69], where the authors show how LRP can be theoretically justified as a deep Taylor decomposition.

## Results

Freezing proved difficult to elicit in front of the cameras. FOG was provoked for ten of the fourteen freezers during the test period, but only seven patients froze in visibility of the cameras. Most freezing episodes occurred during directional change, i.e. after initiating the 180 or 360-degree turn. Subject 1 froze eighteen times, subject 2 thirteen times, subject 3 seven times, subject 4 three times, subject 5 five times, subject 6 nine times, and subject 7 froze once, amounting to a total of fifty-six freezing episodes. The CNN model and the SVM baseline showed excellent classification accuracy. For the fourteen PD patients with FOG, both models achieved comparable accuracy (p = 0.56), with an accuracy of 86.8% and 85.9% by the CNN and SVM, respectively. Interestingly, an analysis of the false detection shows that the lower sensitivity of the CNN is attributed to subject five, for whom all strides were falsely predicted as FGC. Furthermore, most false FGC detections of both models are attributed to subject thirteen and fourteen, two of the three patients that froze during the test period, but not in front of the cameras. For the PD patients without FOG and healthy control subjects, a total of 2421 and 2258 strides were extracted, respectively. For these subjects, the CNN proved the most robust (p = 2.40e-07), with only 26 strides falsely classified for the PD patients without FOG and only a single stride falsely classified for the healthy control subjects. All the results are summarized in Table 3.

**Table 3:** Results of the convolutional neural network (CNN) and support vector machine with linear kernel (LSVC)

Subject Number	CNN	LSVC
1* (FOG: 18, FGC:15)	90.9	90.9
2* (FOG: 13, FGC:9)	72.7	63.6
3* (FOG: 7, FGC:6)	100	100
4* (FOG: 3, FGC:3)	83.3	83.3
5* (FOG: 5, FGC:5)	50.0	70.0
6* (FOG: 9, FGC:9)	100	94.4
7* (FOG: 1, FGC:1)	100	100
8 (FGC: 10)	100	100
9 (FGC: 6)	100	100
10 (FGC: 7)	100	100
11 (FGC: 9)	100	100
12$\dagger$ (FGC: 11)	100	81.8
13$\dagger$ (FGC: 8)	62.5	62.5
14$\dagger$ (FGC: 9)	55.6	55.6
Mean accuracy ± SD	86.8 ± 18.7	85.9 ± 16.5
Sensitivity	82.1	85.7
Specificity	88.9	84.3
PPV	79.3	73.8
NPV	90.6	91.9
Non-freezers (FGC: 2421)	97.6	95.8
Controls (FGC: 2258)	99.9	99.9
Mean accuracy ± SD	98.7 ± 1.66	97.9 ± 2.89

All scores are given in terms of accuracy (%), assessing the performance of the DL models (and LSVC) on the fourteen freezers individually (Subject 1–14), with a summarized score for the 2421 and 2258 strides extracted from the fourteen non-freezers and fourteen healthy controls, respectively. For the fourteen freezers, the performance is additionally assessed in terms of the sensitivity (%), specificity (%), positive predictive value (PPV) (%), and negative predictive value (NPV) (%). The asterisk (*) is used to denote the seven freezers that froze during the protocol. The dagger ($\dagger$) is used to denote the three freezers that froze off camera. The rounded brackets denote the number of extracted strides. For the fourteen freezers, the number of extracted FGCs were controlled for protocol and class imbalance, as explained in the procedure

Mean attribution plots were obtained for six of the seven freezers who experienced FOG during the protocol (Fig. 2a), with the excluded subject for which the model did not perform well (subject five) discussed separately (Fig. 2b), and the fourteen non-freezers and fourteen healthy control subjects (Fig. 2c). The attribution plots visualize the gait characteristics that were the most relevant to the prediction. The mean and standard deviation of the time normalized and re-scaled hip, knee, and ankle joint trajectories in the sagittal plane are plotted and colorized with the relevance map (heatmap) $\sum _{x} R_{x}^{(1)}$ from $\epsilon$-LRP. Positive relevance (red) indicates contribution to FOG, while negative relevance (blue) indicates contribution to FGC.

The attribution analysis of the freezers (Fig. 2a) indicates that the most relevant kinematic features that characterize the movement preceding FOG are the fixed knee extension during the stance phase, reduced peak knee flexion during the swing phase, and fixed ankle dorsiflexion during the swing phase. For FGC, the most relevant features are the peak hip extension and peak knee flexion during the swing phase.

An attribution plot of subject five (Fig. 2b) was created to assess if the heatmaps can uncover an explanation for the poor predictive performance on this subject. Subject five contributed 5 pre-FOG and FGC pairs, with the model classifying all strides as FGC. The lower extremity kinematics indicate that this subject has a severely stooped posture, characterized by large hip and knee flexion. The attribution analysis highlights a near-complete absence of features with a positive contribution to pre-FOG. Additionally, the analysis highlights that the large hip and knee flexion apparent during both pre-FOG and FGC are features that contribute to FGC, indicating that the gait characteristics that uniquely describe this subject are utilized to wrongly classify pre-FOG as FGC.

The attribution analysis of the non-freezers and healthy controls (Fig. 2c) indicates a near complete absence of features with a positive contribution to FOG. The most relevant features to classify FGC for this cohort of subjects are the peak hip and knee flexion during the swing phase.

## Discussion

To tackle the problem of explainable freezing of gait (FOG) prediction, this paper proposed a two-stage pipeline of: (1) a convolutional neural network (CNN) to model the dramatic reduction of movement present before a FOG episode, and (2) layer-wise relevance propagation (LRP) to visualize the underlying features that the CNN perceives as important to model the pathology. The CNN was trained end-to-end on a dataset that consists of fourteen PD patients with FOG. The patients were instructed to complete a FOG provoking protocol of 180 and 360-degree turning, with or without a verbal cognitive dual-task. FOG proved difficult to elicit, with a total of 56 FOG episodes provoked to train the models. This phenomenon is not uncommon, with previous literature also reporting low numbers of freezing episodes occurring in experimental situations, pointing to the unpredictability of FOG [70]. Based on these 56 episodes, a training dataset was created which consists of the time normalized gait cycles directly preceding FOG, each matched with one functional gait cycle (FGC) of the same subject and one FGC of one of the seven NoLab-freezers that did not freeze during the experiments. Despite the relatively low amount of FOG and FGC matched pairs in the training dataset, this study confirms that the dramatic reduction of movement present before freezing can be accurately modelled with DL. After training the CNN to separate movement preceding FOG from normal functional gait, heatmaps were created with LRP. These heatmaps provide insight into the model predictions by quantifying the contribution of each joint trajectory at a certain percentage of the gait cycle to the classification prediction.

From a machine learning perspective, direct comparisons with other studies that researched the motor patterns that precede FOG is challenging because of different underlying study designs. For example, in [29, 71], and [72] the authors extracted time domain and frequency domain features from inertial sensors. Next, the extracted features were used to train a linear discriminant analysis classifier [29], ensemble classifiers [71], or a SVM [72]. In [29] the authors additionally quantitatively assessed the statistical significance of the extracted features. In contrast, DNNs extract features automatically from the raw input signal. To identify whether these features are based on noise or on meaningful kinematic patterns, a qualitative assessment is performed by using heatmap-based attribution methods. To the best of our knowledge, no studies have either: (1) trained a DNN on MoCap-based kinematic data to model the movement that precedes FOG, or (2) used an attribution method to gain insight into a DNNs ability to identify meaningful kinematic patterns that precede FOG.

From a clinical perspective, in [73] the authors found that prior to freezing subjects had severely decreased range of motion in the sagittal plane joint trajectories (with the reduction in the range of motion varying between 31% and 61.5%) of the hip, knee, and ankle. In the interpretability case study, the heatmaps indicated that the CNN model also identified the reduced range of motion as a relevant feature to model the movement preceding FOG. This finding supports the notion that DNN decisions are based on meaningful features. For one of the seven freezers, the CNN was unable to model the movement preceding FOG. The heatmaps indicated that the stooped posture, characterized by a dramatic increase in knee and hip flexion, were the features that the CNN model used to wrongfully classify FOG as FGC. This finding supports the notion that heatmap-based visualizations can aid in uncovering an indication of which features a DNN wrongfully associates with the underlying pathology and thereby allow machine learning practitioners to assess the generalization of their models. Interestingly, the heatmaps also suggest that FOG affects the stance limb to a sufficient degree to influence the prediction, with the fixed knee extension during the stance phase seen as a relevant feature. In [73] the authors only considered FOG events that occurred without directional change. Therefore, future quantitative research should assess whether the stance limb influencing the model predictions is due to the different underlying study designs and thus based on a meaningful kinematic pattern or is the result of noise picked up by the model.

This study also has important limitations. Firstly, the interpretability case study uses a heatmap-based visualization of the learned features. The main limitation of heatmap-based visualizations is the lack of ground-truth, which means that the visualizations can solely be qualitatively assessed [46]. Secondly, the interpretability case study applied to FOG prediction is a proof-of-concept and further research is needed to assess generalization to other use-cases in gait analysis. Thirdly, from a modelling perspective, it should be noted that the threshold model of FOG [74] states that freezing is characterized by continuous degradation of the movement pattern until a threshold is reached and the FOG episode occurs. In this study, the movement preceding FOG is modelled based on the kinematics of a single gait cycle. Therefore, better predictive performance may be achieved by modelling the movement preceding FOG as a sequence of gait cycles, rather than treating each gait cycle as conditionally independent. However, a larger pool of participants with a more varied FOG-provoking protocol will be required to verify this hypothesis. Lastly, the small cohort of PD patients with FOG in this study may not be representative of all freezers, making the conclusions here generalizable to only a small subset of PD patients with FOG.

## Conclusions

Due to the black-box nature of deep learning, clinical gait analysis applications tend to avoid DNNs and retreat to simpler and more interpretable techniques. Using the use-case of FOG prediction, this paper proposed a two-stage pipeline of: (1) a CNN to model the dramatic reduction of movement present before FOG, and (2) LRP to visualize and interpret the underlying features that the CNN perceives as important to the respective classification. The proposed methodology shows that CNNs are capable of modelling the dramatic reduction of movement present before FOG. More importantly, this paper confirms the notion that model interpretation is a powerful tool that allows detailed insight into the complex intertwining between DNN predictions and FOG.

In conclusion, it can be established that the benefit of the proposed interpretability pipeline is two-fold: (1) it can assist expert clinical opinion in explaining DNN predictions by visualizing the kinematic features that the model has learned, and (2) it can aid machine learning practitioners in assessing the generalization of their models by ensuring that the predictions are based on meaningful kinematic features. Future work is now possible in which the proposed pipeline can be used as an automated and objective approach to trigger preventive interventions, i.e. the provision of external stimuli, for FOG. In such work, the interpretations will allow: (1) the clinician to motivate the provision of external stimuli, and (2) a detailed assessment of the efficacy of the intervention by visualizing whether the strides following the intervention show reduced relevance for FOG.

## Supplementary Information

**Additional file 1. Table S1**: The evaluated hyperparameter space of the convolutional neural network (CNN).

## Data Availability

The input set was imported and labelled using Python version 2.7.12 with Biomechanical Toolkit (btk) version 0.3 [75]. Deep learning models were trained on an NVIDIA Tesla K80 GPU using Python version 3.6.8 and Tensorflow version 1.14 [76]. Hyperparameters were optimized using the Hyperopt python library [77], with the cross-validation splits and SVM implemented with scikit-learn version 0.21.3 [65]. Relevance scores were computed with e-LRP as implemented in the DeepExplain framework version 0.3 [44] and visualized with the bipolar colormap [78]. Plotting scripts were modified from spm1d [79].Statistical analysis was conducted using the mlxtend python library [80]. Due to privacy concerns, the dataset analysed during the current study is not publicly available.

## Abbreviations

FOG: Freezing of gait
FGC: Functional gait cycle
PD: Parkinson’s disease
DL: Deep learning
DNN: Deep neural network
CNN: Convolutional neural network
LRP: Layer-wise relevance propagation
H[MYAMP: Y] Hoehn and Yahr
NFOQ-Q: New Freezing of Gait Questionnaire
SVM: Support vector machine
LinearSVC: Linear Support Vector Classifier
BTK: Biomechanical Toolkit
