# Supplementary material for: Modelling and identification of characteristic kinematic features preceding freezing of gait with convolutional neural networks and layer-wise relevance propagation
Source: BMC Med Inform Decis Mak. 2021 Dec 7;21:341. doi: 10.1186/s12911-021-01699-0 (PMC8650332; doi:10.1186/s12911-021-01699-0)
Supplement: Supplementary file 1 — Additional file 1. Table S1: The evaluated hyperparameter space of the convolutional neural network (CNN). [file 12911_2021_1699_MOESM1_ESM.pdf]

Table 1. Suppl. 1: The evaluated hyperparameter space of the convolutional neural network (CNN).

Hyperparameters	CNN
Size of input vector	101
Number of layers	{1,2}
Number of feature maps	{8:8:64}
Filter size	{7:2:19}
Activation function	ELU [?]
Weight initialization	Glorot normal [?]
Dropout probability	{0, 0.1, 0.3, 0.5}
Batch size	32
Pool size	3
Hyperopt iterations	25
Epochs	100
Initial learning rate	0.01
